# Characterization of Methacrylate-Based Resins Containing Methacryl-Polyhedral Oligomeric Silsesquioxanes (MA-POSS-8)

**DOI:** 10.3390/ma14071680

**Published:** 2021-03-29

**Authors:** Marietta Kreutz, Annette Wiegand, Bogna Stawarczyk, Nina Lümkemann, Marta Rizk

**Affiliations:** 1 Department of Preventive Dentistry, Periodontology and Cariology, University Medical Center Göttingen, 37075 Göttingen, Germany; annette.wiegand@med.uni-goettingen.de (A.W.); marta.rizk@med.uni-goettingen.de (M.R.); 2 Department of Prosthetic Dentistry, University Hospital, LMU Munich, 80539 Munich, Germany; bogna.stawarczyk@med.uni-muenchen.de (B.S.); nina.luemkemann@med.uni-muenchen.de (N.L.)

**Keywords:** dental resin, POSS, properties, bioactivity

## Abstract

The use of functionalized dental adhesives that might prevent degradation of the dentin hybrid layer has been proposed. The aim of the study was to characterize the physicochemical properties and the potential to induce mineral precipitation of methacrylate-based resins containing methacryl-functionalized polyhedral oligomeric silsesquioxane (MA-POSS-8). In total, six different compositions of resins based on bisphenol A glycerolate dimethacrylate (BisGMA, 40 to 60 wt.%), triethylene glycol dimethacrylate (TEGDMA, 5 to 35 wt.%) and 2-hydroxyethyl methacrylate (HEMA, 25 or 35 wt.%) were prepared and infiltrated with 5 wt.% MA-POSS-8. Unfilled resins served as control. Degree of conversion, viscosity, Martens hardness, indentation modulus, water sorption, and sol fraction were investigated. Polymerized specimens were examined by SEM/EDX for the presence of Ca/P precipitates after immersion in artificial saliva for 28 days at 37 °C. Statistical analysis was performed with two-way ANOVA and Tukey’s post-hoc test (*p* < 0.05). The degree of conversion ranged from 55.0 to 59.8% and was not affected by the addition of MA-POSS-8. Viscosity ranged from 60.0 to 422.3 mPa*s and was not affected by MA-POSS-8 except for one methacrylate-based resin with 60 wt.% BisGMA. Martens hardness and indentation modulus ranged from 161.3 to 138.1 N/mm^2^ and 4.2 to 3.9 kN/mm^2^ and were affected by MA-POSS-8 in only one resin (50 wt.% BisGMA, 25 wt.% TEGDMA, 25 wt.% HEMA). Water sorption was not affected by MA-POSS-8; sol fraction was below the detection limit. Formation of Ca/P precipitates was observed on all specimens of test and control groups. Material properties were not affected adversly by MA-POSS-8 except for slight differences in Martens Hardness, indentation modulus, viscosity, in some groups.

## 1. Introduction

In order to prevent the hybrid layer between a tooth and composite filling from its typical degradation, several attempts have been made to develop bioactive adhesives that would help to restore the hybrid layer during the degradation process [1,2]. Several bioactive particles were applied in dental resins or adhesives and studied for such applications, however often for the costs of mechanical or other properties. Promising bioactive potential with consistent or improved mechanical properties of the resin could be shown with MA-POSS-8 nanoparticles (mean size 1 to 2 nm) as fillers [3,4,5,6]. MA-POSS-8 particles consist of an inorganic core from silicon and oxygen from which eight methacryl chains originate. The functionalization with the methacrylic chains facilitates further synthesis with the polymer matrix. In addition, the hybrid-like structure of the molecule allows a good distribution in organic substances and might improve the crosslinking density of the polymer [3,7]. Inducing mineral precipitation by MA-POSS-8 are assumed to base on a hydrolysis of the Si–O groups. These Si–O compounds can hydrolyze to Si–OH groups and thereby serve as nucleation point for Ca/P precipitates. In addition, the hybrid-like structure supports a good distribution in organic substances, and thus may also lead to a better network density [4].

POSS were successfully used to improve the mechanical properties of dental composites [3,8,9]. Rizk et al. [4] were able to show Ca/P precipitation on the surface of the specimens after adding 10 wt.% MA-POSS-8 to a conventional dental adhesive, unlike in the control, particle-free group. The addition of MA-POSS-8 also did not worsen material properties such as viscosity or conversion degree, and even reduced water sorption. In another study by Rizk et al. [6] it was observed that the addition of 10 wt.% MA-POSS-8 to an conventional adhesive leads to a lower degree of conversion and a reduced shear bond strength compared to concentrations of 2 and 5 wt.%. Due to the better material properties and the simultaneous mineralization potential of 5 wt.% MA-POSS-8 compared to 10 wt.%, a concentration of 5 wt.% MA-POSS-8 was selected for this study. However, due to the unclear composition of the commercial adhesives and bondings, the interaction and reactions of the fillers in these materials cannot be fully understood. Commercially available adhesives consist of a variety of components that can form potential interactions and chemical bonds with each other or with the MA-POSS-8 particles. However, the potential effect of MA-POSS-8 can only be well understood if the composition of the adhesive/bonding is known. BisGMA, TEGDMA, and HEMA are the most commonly used methacrylates in dental adhesives and bondings and experimental dental resin blends [1,10,11,12,13,14]. The aim of this study is therefore to investigate the physicochemical properties and the potential to induce mineral precipitation of MA-POSS-8 using six methacrylate-based resins consisting of BisGMA, TEGDMA, and HEMA. The null hypothesis was that there were no significant differences in the physicochemical properties and the potential to induce mineral precipitation in experimental resins with and without MA-POSS-8.

## 2. Materials and Methods

### 2.1. Experimental Methacrylate-Based Resins

In total, six experimental methacrylate-based resins were prepared consisting of the monomers BisGMA ([H_2_C=C(CH_3_)CO_2_CH_2_CH(OH)CH_2_OC_6_H_4_]_2_C(CH_3_)_2_, CAS: 1565-94-2, Sigma–Aldrich Chemistry, St. Louis, MO, USA), TEGDMA (CH_2_=C(CH_3_)COO(CH_2_CH_2_O)_3_COC(CH_3_)=CH_2_, CAS-number 109-16-0, Sigma–Aldrich Chemistry), and HEMA (CH_2_=C(CH_3_)COOCH_2_CH_2_OH, CAS: 868-77-9, Sigma–Aldrich Chemistry, Table 1). Camphorchinone and dimethylamino-methacrylate (Sigma–Aldrich Chemistry), each 0.5 wt.%, were used as photo-initiators. The resins were mixed at room temperature with a magnetic stirrer for 10 min in a dark environment to avoid polymerization. Afterwards, 5 wt.% of MA-POSS-8 (Hybrid Plastics^®^, Hattiesburg, MS, USA) was added to the experimental groups, while the control groups remained unchanged. All resins were then further mixed for 10 min in dark. The resin blends for measuring water sorption, sol fraction, mineral inducing capacity, Martens hardness, and indentation modulus were then removed with a pipette and placed in a Teflon mold. Subsequently, each specimen was light cured from each side for 60 s (>1000 mW/cm^2^, Bluephase, Ivoclar Vivadent GmbH, Schaan, Liechtenstein). To examine the degree of conversion the resin blends were immediately used. For viscosity measurements resins were prepared without an initiator.

### 2.2. Degree of Conversion

Fourier transformation infrared spectrometry equipped with an attenuated total reflectance accessory (FTIR-ATR, Bruker, Billerica, MA, USA) was used to determine the degree of conversion (n = 5). Absorbance spectra were collected by 20 repeated scans in the range of 700 to 4000 cm^−1^ with a resolution of 4 cm^−1^. Before each experiment, a background spectrum was measured and subtracted from the following spectrum. A drop of the uncured resin was applied on the diamond crystal. The crystal area was fully covered by the adhesive. This reduces the effect of possible oxidation during the measurements. After 4 repeated measurements of the uncured adhesive of 120 s, the sample was cured for 60 s (>1000 mW/cm^2^, Bluephase, Ivoclar Vivadent GmbH, Schaan, Liechtenstein) and the spectra were collected for the next 390 s.

The ratio between the peak heights of the vinyl- (aliphatic C=C 1638 cm^−1^) and the benzyl-groups (aromatic C=C 1608 cm^−1^) before and after curing was used to calculate the degree of conversion [4]:

(1) Degree of conversion (%)=(1−(PVinylPBenzyl)cured(PVinylPBenzyl)uncured) × 100

$P_{Vinyl}:$ Height of the peak of vinyl group
$P_{Benzyl}:$ Height of the peak of benzyl group

### 2.3. Viscosity

The oscillation tests (n = 3) were performed at 25 °C using a rheometer (AR-G2 rheometer, TA Instruments, New Castle, New Delaware, DE, USA). A cone plate geometry with 40 mm diameter and 2° cone angle was used. The resin blends were prepared as described above but without an initiator to avoid polymerization during the experiment. Initially, the amplitude was varied (20 rad/s, 5 to 20%) at a constant frequency to confirm the linear regime of the viscoelastic behavior of the samples. Subsequently, the shear test was performed at the constant amplitude of 15% and varying frequency (10 to 100 rad/s and 100 to 5 rad/s). The steady shear was studied in the range from 1 to 100 1/s and from 100 to 1/s. The two runs were used to check for the reproducibility and to prevent the influence of any sample history on the results. The results of the viscosity were fitted by cross-fit model (Newtonian).

### 2.4. Martens Hardness and Indentation Modulus

Cylindrical specimens were prepared in standardized Teflon forms (diameter 8 mm; thickness 2 mm; per group n = 5) and polymerized for 60 s from both sides (>1000 mW/cm^2^, Bluephase, Ivoclar Vivadent GmbH, Schaan, Liechtenstein). After 24 h under dry conditions at room temperature, the samples were embedded in resin (Paladur, Kulzer GmbH, Hanau, Germany) and polished (Tegramin-20, Struers GmbH, Ballerup, Denmark; SiC Foil #1200 200 mm, Struers GmbH).

Measurement of Martens hardness and indentation modulus was performed with a universal testing machine (Zwick/Roell ZHU 0.2/Z2.5, Zwick GmbH & Co. Kg, Ulm, Germany). The Martens hardness (*HM*) was derived from the applied force (*F*) and the impressed area *A_s_* calculated from a penetration depth (*h*) [15]:

(2) $$HM=\;\frac{F}{A_{s}\left(h\right)}=\;\frac{F}{26,43\;\times \;h^{2}}$$

The indentation modulus E_IT_ can be determined from the tangent for the calculation of indentation hardness.

(3) $$E_{\mathrm{IT}}\;=\;(1-\upsilon_{s}^{2})\;\times \;{\left(\frac{1}{E_{R}}\;-\;\frac{\left(1\;-\;\upsilon_{i}{}^{2}\right)}{E_{i}}\right)}^{-1}\;\mathrm{with}\;E_{R}=\;\frac{\sqrt{\pi }}{\sqrt[{2C}]{Ap}}$$

where $\nu_{S\;}$ is Poisson’s ratio of the test piece (0.35); $\upsilon_{i}$ is Poisson’s ratio of the indenter (0.3); $E_{R}$ is the reduced modulus of the indentation contact; $E_{i}$ is the modulus of the indenter (1.14 × 10^6^ N/mm^2^); C is the compliance of the contact, i.e., d*h*/d*f* is evaluated of the test force removal curve at maximum test force; $\sqrt{A_{p}}$ is $\;4.95*h_{c}$ for the Vickers indenter; *h_c_* is the depth of contact of the indenter with the specimen calculated.

The hardness measurement was performed three times per specimen and averaged.

### 2.5. Water Sorption and Sol Fraction

The specimens (diameter 6 mm; thickness 2 mm, n = 5) were prepared, as described above. After curing (>1000 mW/cm^2^, Bluephase, Ivoclar Vivadent GmbH, Schaan, Liechtenstein), the specimens were kept in a desiccator with silicate gel for drying. The mass was measured with a precision scale (Sartorius, Göttingen) every second day until the difference between two following measurements was below or equal to 0.1 mg ($m_{0}$). Diameter and thickness were measured and the volume ($V_{m_{0}}$) was calculated for each specimen. Afterwards, the specimens were stored in distilled water at 37 °C and weighted every second day until they reached a steady value ($m_{1}$). Then they were dried again at the desiccator to obtain an equilibrated mass $m_{2}$. The following equations were used to determine the water sorption (*WS*) and the sol fraction (SF):

(4) $$WS=\frac{\left(m_{1}-m_{2}\right)}{V_{m_{0}}}$$

(5) $$SF=\;\frac{\left(m_{0}-m_{2}\right)}{V_{m_{0}}}$$

### 2.6. Mineral Precipitation Capacity

Specimens (each group n = 2, diameter 4 mm; thickness 2 mm) were prepared in a teflon form and light cured for 60 s from both sides (>1000 mW/cm^2^, Bluephase, Ivoclar Vivadent GmbH, Schaan, Liechtenstein). Then, the specimens were polished (preparation diamond 837KREF.314.014, Gebr. Brasseler GmbH & Co. KG, Lemgo, Germany) and stored in artificial saliva [16] at 37 °C for 4 weeks while the medium was changed every second to third day. Artificial saliva was composed of 0.00113 mmol/L ascorbic acid (Carl Roth GmbH, Karlsruhe, Germany), 0.5 mmol/L glucose (Carl Roth GmbH, Karlsruhe, Germany), 9.9 mmol/L NaCl (Merck KGaA, Darmstadt, Germany), 1.5 mmol/L CaCl_2_*2H_2_O (Merck KGaA, Darmstadt, Germany), 3.0 mmol/L NH_4_Cl (Carl Roth GmbH, Karlsruhe, Germany), 17.0 mmol/l KCl (Carl Roth GmbH, Karlsruhe, Germany), 2.0 mmol/L NaSCN (Carl Roth GmbH, Karlsruhe, Germany), 2.4 mmol/l KH_2_PO_4_ (Carl Roth GmbH, Karlsruhe, Germany), 3.3 mmol/L urea (Carl Roth GmbH, Karlsruhe, Germany) and 2.4 mmol/L Na_2_HPO_4_ (Carl Roth GmbH, Karlsruhe, Germany). Afterwards all specimens were carefully rinsed with distilled water and stored in a desiccator. One specimen of each group was sputtered by platinum-palladium and inspected by scanning electron microscopy at 10 kV for possible precipitates (Ultra Plus, Carl Zeiss GmbH, Jena, Germany). To determine the elemental composition of the precipitates, energy-dispersive X-ray spectroscopy analyses at 15 kV was performed (Quanta 200 F, FEI Company, Hillsboro, OR, USA) at three characteristic points on carbon sputtered specimens (each group n = 1).

### 2.7. Statistical Analysis

Degree of conversion, water sorption, viscosity, Martens hardness and indentation modulus were evaluated by two-way ANOVA, factors being the kind of experimental resin and MA-POSS-8 and Tukey’s post-hoc test (Statistica, version 13.3, StatSoft Europe GmbH, Hamburg, Germany). *p*-values < 0.05 were considered statistically significant.

## 3. Results

### 3.1. Degree of Conversion

The degree of conversion ranged from 55.0 to 59.8% (Table 2). ANOVA revealed no effect of the composition of experimental methacrylate-based resin (*p* < 0.101), but the presence of MA-POSS-8 significantly affected the results (*p* < 0.001). No interaction between the factors was observed (*p* < 0.450). Pairwise comparisons between resins with and without MA-POSS-8 revealed no significant differences in the different groups (Table 3).

### 3.2. Viscosity

Both parameters, the composition of experimental methacrylate-based resin (*p* < 0.001) and the presence of MA-POSS-8 (*p* < 0.001) significantly affected viscosity; the interaction was significant (*p* < 0.0001). Only in resin 3, the viscosity decreased from 422.3 to 333.9 mPa*s when MA-POSS-8 was added. A tendency to lower viscosity through the MA-POSS-8 can be seen in all studied groups except resin 4; however, the pairwise comparisons revealed no significant differences.

### 3.3. Martens Hardness and Indentation Modulus

The monomer composition of experimental resin blends showed a significant effect on Martens hardness (*p* < 0.001) and indentation modulus (*p* < 0.001). In addition, a significant effect of MA-POSS-8 could be shown for Martens hardness (*p* < 0.001) and indentation modulus (*p* < 0.001). For both Martens hardness (*p* < 0.001) and indentation modulus (*p* < 0.003) the interaction between both factors was significant. Martens hardness was reduced significantly only in resin 2 from 161.3 (without MA-POSS-8) to 138.1 N/mm^2^ (with MA-POSS-8). Similarly, the addition of MA-POSS-8 resulted in a significant difference in the indentation modulus in resin 2 (Table 2).

### 3.4. Water Sorption and Sol Fraction

Water sorption was significantly influenced by experimental resin composition (*p* < 0.001) and MA-POSS-8 (*p* < 0.002), but no significant interaction between the factors was observed (*p* < 0.540). Post-hoc tests showed no significant differences between the control and test groups. The lowest water sorption was found in the groups with the lowest amount of HEMA and the highest amount of BisGMA; and oppositely, the most hydrophilic behavior was detected in groups with the highest proportion of HEMA and lowest concentration of BisGMA. By keeping a constant proportion of BisGMA, water sorption increased with increasing HEMA concentration (Table 2).

Sol fraction was below the detection limit in all adhesives and was therefore not further evaluated.

### 3.5. Mineral Precipitation Capacity

**Figure 1:**
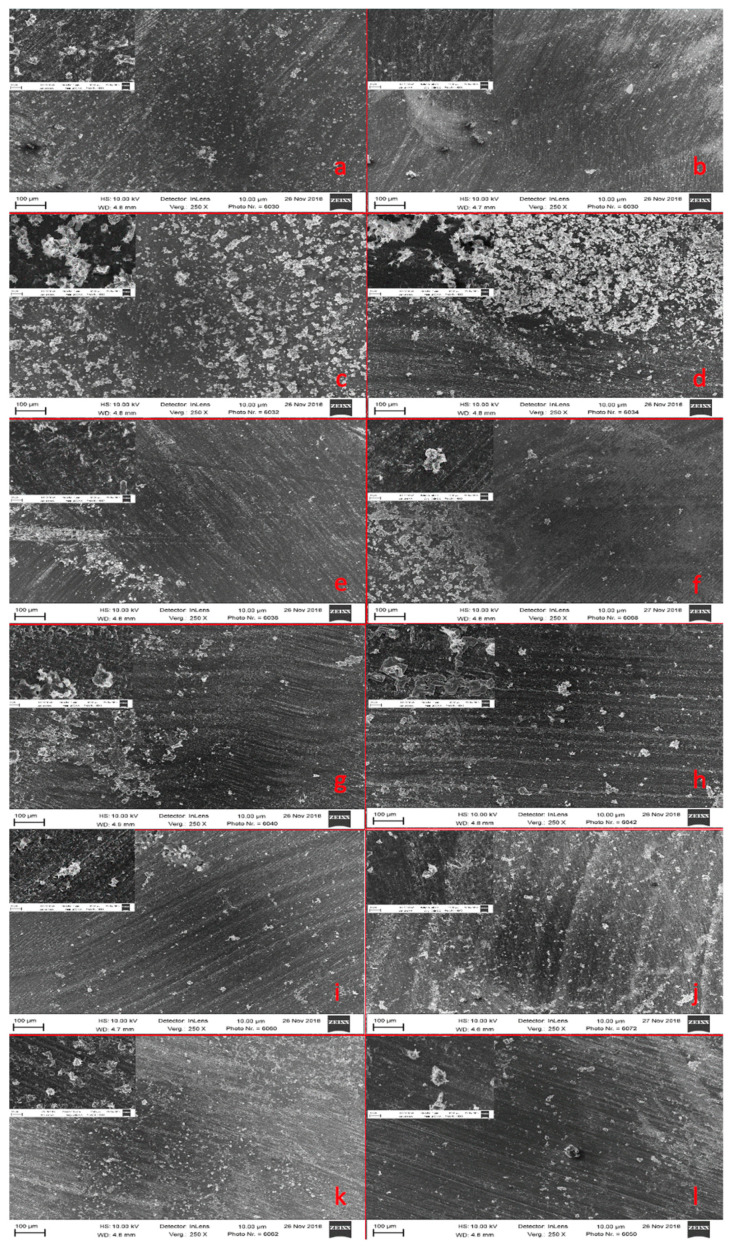
Representative SEM image of the surface of (**a**) the methacrylate-based resin 1 without MA-POSS-8; (**b**) the methacrylate based resin 1 with MA-POSS-8; (**c**) the methacrylate based resin 2 without MA-POSS-8; (**d**) the methacrylate based resin 2 with MA-POSS-8; (**e**) the methacrylate based resin 3 without MA-POSS-8; (**f**) the methacrylate based resin 3 with MA-POSS-8; (**g**) the methacrylate based resin 4 without MA-POSS-8; (**h**) the methacrylate based resin 4 with MA-POSS-8; (**i**) the methacrylate based resin 5 without MA-POSS-8; (**j**) the methacrylate based resin 5 with MA-POSS-8; (**k**) the methacrylate based resin 6 without MA-POSS-8; (**l**) the methacrylate based resin 6 with MA-POSS-8 in 250 and 2500 fold magnification.

In all resin blends, crystalline structures could be observed on the surface of the specimens. Resin 2 showed slightly more precipitates on the surface than the other resins (Figure 1). The EDX analysis confirmed that the precipitates contained calcium and phosphorus (Table 3).

## 4. Discussion

This study investigated the effect of MA-POSS-8 particles on the properties of methacrylate-based resin matrices. As the physicochemical properties predominantly did not differ between experimental resins with and without MA-POSS-8 and the mineral precipitation on resin specimens with and without MA-POSS-8 was not different, the null hypothesis is accepted.

In order to relate the effect of MA-POSS-8 to individual components, six experimental resins were prepared and the physicochemical properties and mineral precipitation capacity depending on the proportion of BisGMA, TEGDMA, and HEMA were analyzed. BisGMA, TEGDMA and HEMA are monomers commonly used in adhesives and bondings [10]. The concentrations of the monomers were varied to investigate the possible interaction of MA-POSS-8 with the polymer matrix of various properties and microstructure.

The increased water sorption with higher HEMA concentration as observed in this study was found by several other studies and can be explained by the strongly hydrophilic character of this monomer [14,17]. By adding 10 wt.% POSS particles to a conventional adhesive, Rizk et al. [4] showed a reduction in water sorption. This is attributed to the hydrophobic character of MA-POSS-8 and the improved network density [4]. In this study, no effect of MA-POSS-8 on water sorption could be determined. This could possibly be due to the lower proportion of MA-POSS-8. The addition of MA-POSS-8 led to a significant reduction from 422.3 to 333.9 mPa*s in viscosity in resin 3, which is the group with the most viscous neat resin. The increasing BisGMA concentration in this group caused higher viscosity due to the high molecular weight and stiffness of BisGMA [18,19]. This viscosity reduction may be mainly due to the relatively low molecular weight and the oligomer shell of the hybrid MA-POSS-8 particles which lead to a solvent-like behavior in highly viscous blends [20]. In resin 2, the addition of MA-POSS-8 led to a significant decrease in Martens hardness and indentation modulus, while the addition of MA-POSS-8 showed no effect in the other resins. So far, no study has examined the influence of the MA-POSS-8 on the Martens hardness of methacrylate-based resins. This characteristic is conventionally used to indirectly investigate the degree of conversion and thus matrix density since it typically increases with increasing degree of conversion [21,22,23].

In this study, the incorporation of MA-POSS-8 did not hinder the polymerization of all experimental resins. This is in line with a previous study by Rizk et al. [4]. Due to the molecular structure of MA-POSS-8 and its high functionality it can act as an important crosslink point and thus improve the actual network density, even if only partially reacted [24]. At the same time, the residual unreacted C=C groups could worsen the apparent degree of conversion [4]. Considering the same reactivity of all monomers in the systems with and without MA-POSS-8, an approximated degree of conversion of the particles (*DC_POSS_*) can be estimated from the measured *DC* of the unfilled (*DC_Adh_*) and filled (*DC_AdhPOSS_*) adhesive system:

(6) $$DC_{POSS}=\frac{1}{V_{AdhPOSS}}\left(DC_{AdhPOSS}-DC_{Adh}V_{Adh}\right)$$

where *VAdh* and *VAdhPOSS* are the normalized molar fractions of C=C groups on the only monomers resp. MA-POSS-8 particles in the system. These were estimated based on each adhesive composition (Table 1) and the molecular weight of the reactants (MA-POSS-8 1433.97 mol/g, BisGMA 512.59 mol/g, TEGDMA 286.32 mol/g, HEMA130.14 mol/g). The ideal number of functional groups, *f_x_,* was considered for this estimation (*f*_MA-POSS-8_ = 8, *f*_BisGMA_ = 2, *f*_TEGDMA_ = 2, and *f*_HEMA_ = 1).

The calculated *DC_POSS_* ranged from 3.1% up to 56.4%. In the groups with a higher concentration of HEMA (resins 4 to 6), a decreasing trend with an increase of BisGMA fraction and thus also viscosity of the adhesive was observed (*DC_POSS4_* = 56.4%, *DC_POSS5_* = 17.1%, *DC_POSS6_* = 9.6 %). This trend is not seen in the group with lower HEMA fraction (*DC_POSS1_* = 3.1%, *DC_POSS2_* = 49.5%, *DC_POSS3_* = 11.6 %). Interestingly, the largest difference was found in the groups with only a slight difference in the monomer composition and a very similar viscosity (resins 1 and 4). In this case, a higher HEMA proportion leads to a higher *DC_POSS_*, possibly due to a dilution effect and increase of mobility of radicals. Additionally, at both adhesives with the highest viscosity (3 and 6) a very low conversion of MA-POSS-8 was estimated. Overall, there are certainly several factors that influence the MA-POSS-8 conversion. Our data indicate the importance of viscosity and proportion of monomers with various functionality and size. Since in all groups a positive *DC_POSS_* was determined, the conversion of all reactive components and the network density could be stable or even improved through the addition of MA-POSS-8 even though such conclusion could not be taken based barely on the measured *DC* or *E_IT_*.

In this study, the addition of MA-POSS-8 did not improve the properties of the experimental resins. Other studies investigating the effect of MA-POSS-8 in concentrations below 5% found significantly improved mechanical properties [3,24], while higher concentrations (≥10 wt.%) led to a reduction of the mechanical properties [24,25,26]. However, methodological differences between the studies must be considered, as the effect of MA-POSS-8 was often investigated in resins that contained other fillers, e.g., barium glass or silicone dioxide, to simulate a dental composite [3,8,24]. Possible interactions among different fillers need to be investigated.

After 28 days of storage in artificial saliva Ca/P-precipitation could be observed on the surface of the test and control groups, without differences. A similar observation was described by Engstrand et al. [5], who investigated the mineral inducing capacity of POSS mixed with polyethylene glycol (PEG). There, precipitates were also detected on the samples from pure PEG. PEG is able to form a complex with divalent metal ions, such as calcium, causing the formation of precipitates. Possibly, the formation of precipitates on the specimens of the control group is affected by composition of the polymer matrix. TEGDMA and HEMA possess free responsive OH groups which might form precipitates with ions (e.g., Ca^2+^) from the medium, serving as nucleation points.

Similarly, EDX analysis of the precipitates was below the stochiometric molar ratio of hydroxyapatite (1.67) at any resin. This ratio is preferred since it defines the structure of the natural enamel. However, it should be noted that the structure of the Ca/P precipitates is highly depend on the composition, time of immersion and the pH of the medium used [27,28].

## 5. Conclusions

The addition of MA-POSS-8 to the methacrylate-based resins did not affect the material properties adversely. Depending on the monomer concentrations, MA-POSS-8 may slightly improve viscosity, or slightly affect other mechanical properties. To improve mineral precipitation and capacity, probably a concentration higher than 5 wt.% MA-POSS-8 is needed.

## Figures and Tables

**Table 1:** Monomer composition of the studied methacrylate-based resins in wt.%.

Methacrylate-Based Resin	BisGMA	TEGDMA	HEMA
1	40	35	25
2	50	25	25
3	60	15	25
4	40	25	35
5	50	15	35
6	60	5	35

**Table 2:** Mean ± standard deviations of degree of conversion (DC), viscosity (η), Martens hardness (HM), indentation modulus (E_IT_) and water sorption (WS) of the experimental metharcylate-based resins with and without MA-POSS-8.

Methacrylate-Based Resin	MA-POSS-8 (wt%)	DC (%)	η (mPa*s)	HM (N/mm^2^)	E_IT_ (kN/mm^2^)	WS (µg/mm^3^)
1	0	58.2 ± 0.4 ^a^	66.2 ± 2.6 ^d^	149.1 ± 9.5 ^ab^	4.0 ± 0.1 ^ab^	105.4 ± 7.5 ^bc^
	5	55.6 ± 2.1 ^A^	65.9 ± 0.9 ^C^	144.0 ± 6.9 ^A^	3.9 ± 0.1 ^A^	101.6 ± 7.9 ^BC^
2	0	57.6 ± 4.3 ^a^	146.0 ± 3.3 ^c^	161.3 ± 2.3 ^a^*	4.2 ± 0.1 ^a^*	100.5 ± 5.9 ^bc^
	5	57.2 ± 2.2 ^A^	136.9 ± 1.4 ^B^	138.1 ± 2.5 ^AB^*	3.9 ± 0.0 ^A^*	92.7 ± 6.9 ^C^
3	0	57.4 ± 0.9 ^a^	422.3 ± 16.8 ^a^*	137.7 ± 7.9 ^b^	4.0 ± 0.1 ^ab^	93.9 ± 10.6 ^c^
	5	55.0 ± 0.7 ^A^	333.9 ± 24.7 ^A^*	151.4 ± 4.7 ^A^	4.1 ± 0.1 ^A^	86.0 ± 5.5 ^C^
4	0	56.4 ± 1.8 ^a^	60.0 ± 0.8 ^d^	110.4 ± 14.6 ^c^	3.4 ± 0.3 ^c^	135.7 ± 13.2 ^a^
	5	56.4 ± 1.5 ^A^	62.1 ± 0.2 ^C^	101.4 ± 6.9 ^C^	3.3 ± 0.2 ^B^	122.9 ± 8.0 ^A^
5	0	57.8 ± 1.3 ^a^	137.3 ± 3.5 ^c^	156.8 ± 9.0 ^a^	4.2 ± 0.2 ^a^	115.2 ± 7.9 ^b^
	5	55.8 ± 2.2 ^A^	133.9 ± 1.5 ^B^	147.4 ± 5.3 ^A^	3.9 ± 0.1 ^A^	109.5 ± 22.1 ^AB^
6	0	59.8 ± 1.1 ^a^	338.7 ± 25.1 ^b^	119.1 ± 7.0 ^c^	3.7 ± 0.2 ^bc^	112.4 ± 9.8 ^b^
	5	57.2 ± 0.4 ^A^	312.3 ± 2.1 ^A^	126.6 ± 4.7 ^B^	3.8 ± 0.2 ^A^	112.6 ± 13.1 ^AB^

Significant differences between the experimental methacrylate-based resins 1-6 without the addition of MA-POSS-8 are marked by lowercase letters. Significant differences between the experimental methacrylate-based resins 1-6 with the addition of MA-POSS-8 are marked by capital letters. Significant differences between the control and test group within one experimental methacrylate-based resin are marked with *.

**Table 3:** Mean ± standard deviations of the Ca/P ratio of the experimental methacrylate-based resins with and without MA-POSS-8.

Methacrylate-Based Resin	MA-POSS-8 (wt.%)	Ca/P Ratio
1	0	0.40 ± 0.07
	5	0.73 ± 0.22
2	0	0.80 ± 0.24
	5	1.12 ± 0.06
3	0	1.30 ± 0.05
	5	0.54 ± 0.25
4	0	0.47 ± 0.08
	5	1.12 ± 0.24
5	0	0.88 ± 0.19
	5	1.29 ± 0.06
6	0	1.12 ± 0.03
	5	0.55 ± 0.07

## Data Availability

Data is contained within the article.
